# Simulations inform design of regional occupancy‐based monitoring for a sparsely distributed, territorial species

**DOI:** 10.1002/ece3.3725

**Published:** 2017-12-20

**Authors:** Quresh S. Latif, Martha M. Ellis, Victoria A. Saab, Kim Mellen‐McLean

**Affiliations:** ^1^ Rocky Mountain Research Station U.S. Forest Service Bozeman MT USA; ^2^ Montana State University Bozeman MT USA; ^3^ Region 6 U.S. Forest Service Portland OR USA

**Keywords:** broad‐scale monitoring, detection probability, *Picoides albolvartus*, population trends, power analysis, spatial simulation, species conservation, survey design, white‐headed woodpecker

## Abstract

Sparsely distributed species attract conservation concern, but insufficient information on population trends challenges conservation and funding prioritization. Occupancy‐based monitoring is attractive for these species, but appropriate sampling design and inference depend on particulars of the study system. We employed spatially explicit simulations to identify minimum levels of sampling effort for a regional occupancy monitoring study design, using white‐headed woodpeckers (*Picoides albolvartus*), a sparsely distributed, territorial species threatened by habitat decline and degradation, as a case study. We compared the original design with commonly proposed alternatives with varying targets of inference (i.e., species range, space use, or abundance) and spatial extent of sampling. Sampling effort needed to achieve adequate power to observe a long‐term population trend (≥80% chance to observe a 2% yearly decline over 20 years) with the previously used study design consisted of annually monitoring ≥120 transects using a single‐survey approach or ≥90 transects surveyed twice per year using a repeat‐survey approach. Designs that shifted inference toward finer‐resolution trends in abundance and extended the spatial extent of sampling by shortening transects, employing a single‐survey approach to monitoring, and incorporating a panel design (33% of units surveyed per year) improved power and reduced error in estimating abundance trends. In contrast, efforts to monitor coarse‐scale trends in species range or space use with repeat surveys provided extremely limited statistical power. *Synthesis and applications*. Sampling resolutions that approximate home range size, spatially extensive sampling, and designs that target inference of abundance trends rather than range dynamics are probably best suited and most feasible for broad‐scale occupancy‐based monitoring of sparsely distributed territorial animal species.

## 1 INTRODUCTION

Population monitoring informs biological conservation by revealing population trends, which inform conservation status and funding priorities (Marsh & Trenham, 2008). Conservationists focus on species experiencing severe or consistent declines due to anthropogenic impacts that elevate extinction risk (Male, Bean, & Schwartz, 2005; Rodrigues, Pilgrim, Lamoreux, Hoffmann, & Brooks, 2006). Species of uncertain status due to insufficient data are difficult to target, even if life history or declining habitat warrant concern. Information for prioritizing conservation is particularly limited for sparsely distributed species (Roberts, Taylor, & Joppa, 2016). Imperfect detectability and difficulties with modeling also impose challenges for territorial animals (Efford & Dawson, 2012; Latif, Ellis, & Amundson, 2016). Low detectability and an extensive range may necessitate broad and sustained effort to characterize population status, despite typically limited funding (Joseph, Field, Wilcox, & Possingham, 2006).

Biologists increasingly use occupancy‐based monitoring for these species (Ellis, Ivan, & Schwartz, 2014; Joseph et al., 2006). Detection–nondetection data demand less funding than counts or mark–recapture data, allowing more spatially extensive surveys (Joseph et al., 2006; Noon, Bailey, Sisk, & McKelvey, 2012); while replicate sampling can correct for imperfect detection (MacKenzie et al., 2002; Tyre et al., 2003). Occupancy quantifies species distribution, which can inform species range at coarse scales or finer‐scale changes in space use or abundance, all relevant to extinction risk (Clare, Anderson, & MacFarland, 2015; Joseph et al., 2006; Noon et al., 2012).

Study design for monitoring occupancy depends on desired inference and species ecology. Relatively large sampling units potentially occupied by multiple individuals can efficiently inform species range estimates, whereas smaller units may be better for tracking finer‐scale changes in local abundance (Clare et al., 2015; Efford & Dawson, 2012; Noon et al., 2012). With smaller units, the timing of replicate samples used to correct for detectability in relation to territorial movement further shapes potential inference (Efford & Dawson, 2012; Latif et al., 2016; Valente, Hutchinson, & Betts, 2017). Sampling continuously distributed populations of mobile individuals with indefinite home range boundaries is especially challenging; such populations are inherently heterogeneous in ways not quantified by commonly used models, potentially obscuring inference (Efford & Dawson, 2012). More complex models that correctly specify this heterogeneity typically require more sampling effort, which may be infeasible or compromise sampling extent needed to document broad‐scale trends (Welsh, Lindenmayer, & Donnelly, 2013). Simulation approaches can help inform design of occupancy‐based monitoring with such inherent and unavoidable misspecification of spatial heterogeneity (Ellis, Ivan, Tucker, & Schwartz, 2015; Ellis et al., 2014).

Desired inference should primarily determine monitoring approach, but pragmatic considerations also influence study design. Biologists may size sampling units for study area coverage or to match the resolution of available environmental data (Zielinski, Baldwin, Truex, Tucker, & Flebbe, 2013; e.g., Steenweg et al., 2016; but see Linden, Fuller, Royle, & Hare, 2017). Additionally, biologists select statistical models that best leverage available data. For example, despite a fundamental relationship between detectability and abundance (Royle & Nichols, 2003), analysts may hold detectability constant for parsimony (e.g., Zielinski et al., 2013). Sampling is often then designed to achieve adequate statistical power for tracking occupancy trends without a priori specifying desired targets of inference (e.g., species range, space use, or abundance). Inference of process, however, is ultimately needed to inform conservation.

**Figure 1:**
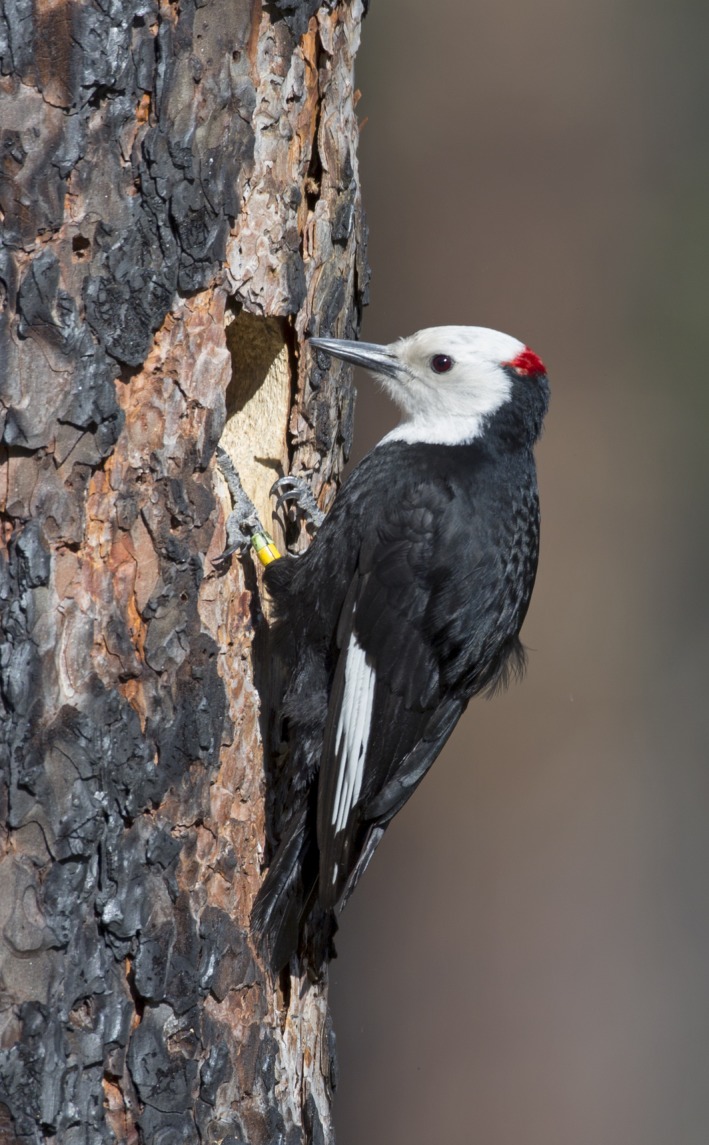
Photograph of a White‐headed Woodpecker

Our questions on monitoring design were motivated by a regional occupancy‐based monitoring program for white‐headed woodpecker (*Picoides albolvartus*; hereafter WHWO; Figure 1), a sparsely distributed, regionally endemic species with narrow habitat requirements (Garrett, Raphael, & Dixon, 1996; Latif, Saab, Mellen‐Mclean, & Dudley, 2015). WHWO depend on dry mixed conifer forests dominated by ponderosa pine (*Pinus ponderosa*) and maintained by mixed‐severity fire (cf. Hessburg, Agee, & Franklin, 2005). Recent habitat declines and evidence of low reproductive success in some areas have raised conservation concerns (Hollenbeck, Saab, & Frenzel, 2011), but data on population trends are lacking (Wisdom et al., 2002).

**Figure 2:**
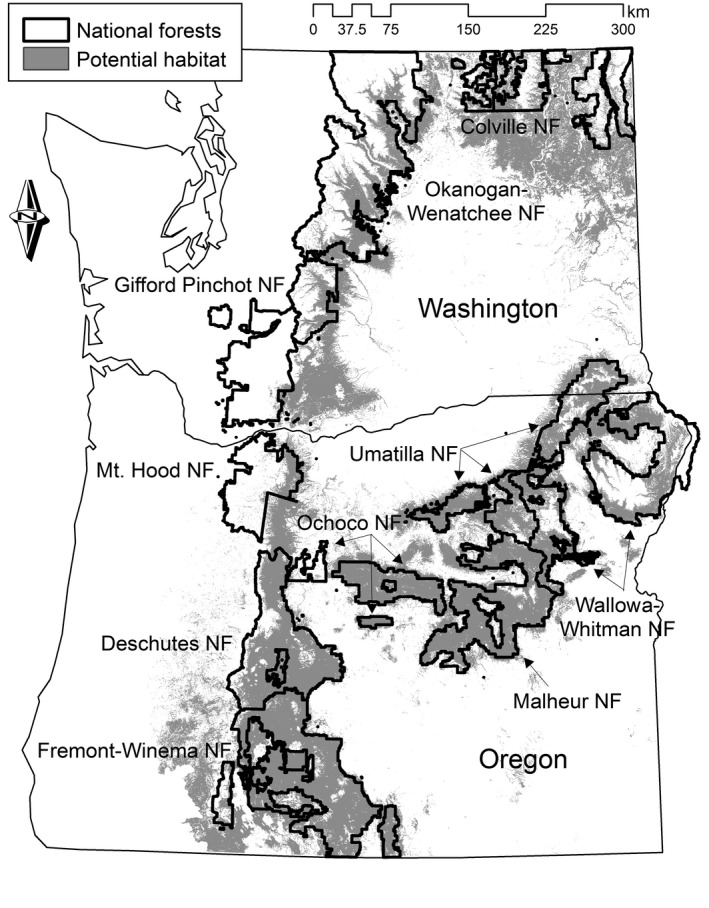
National forests of the eastern Cascade Mountains, Oregon and Washington, U.S.A. White‐headed Woodpecker regional monitoring focused on potential habitat (gray), where large‐cone pine species (mainly ponderosa) dominate

To help fill this information gap, regional occupancy‐based monitoring was established to evaluate population and distributional trends (Mellen‐McLean, Saab, Bresson, Wales, & VanNorman, 2015). Repeat detection–nondetection surveys along transects in potential habitat of Oregon and Washington (Figure 2) informed occupancy trends corrected for imperfect detection. Surveyors applied a common protocol for birds of point‐based surveys oriented along transects (see also Rota, Fletcher, Dorazio, & Betts, 2009; Valente et al., 2017). Available funding was substantial (~$800 thousand) but nevertheless limited monitoring to 6 years at 30 transects while also accommodating other objectives (Mellen‐McLean et al., 2015). Growing agency interest in white‐headed woodpeckers could motivate expanded and more focused monitoring of long‐term trends, which we aimed to inform.

To address the questions raised by this case study, we used simulations to evaluate alternative approaches to regional monitoring while explicitly considering potential inference and species ecology. Spatially explicit simulations correctly represented model misspecification typically inherent with occupancy‐based monitoring of continuously distributed populations, improving estimates of statistical power for informing study design (Ellis et al., 2014, 2015). We assessed minimum effort needed for desirable statistical power given the historical study design, and we explored how alternate sampling allocations influenced statistical power. We considered sampling allocations alternately suited for inferring coarse‐scale distributional changes or range dynamics versus finer‐scale changes in abundance or space use (e.g., Valente et al., 2017). Candidate designs varied in how they favored spatially extensive versus more intensive survey allocation, the value of which depends on population heterogeneity (Rhodes & Jonzén, 2011). Alternatives considered here represent commonly used designs for broad‐scale occupancy‐based studies, thus providing general guidance for monitoring sparsely distributed, territorial animals.

## 2 MATERIALS AND METHODS

### 2.1 White‐headed Woodpecker regional monitoring

Occupancy‐based monitoring of WHWO across the inland northwestern United States was originally implemented in 2011–2016 (Mellen‐McLean et al., 2015). Surveys occurred along 30 transects twice a year during the nesting season, May 1–June 30. Surveyors broadcast recorded calls and drumming to elicit territorial responses to improve detectability. Transects were 10 survey points spaced ~300 m apart. A transect survey consisted of surveying each point along a transect. This approach is common for surveying birds (common approach for birds; e.g., Amundson, Royle, & Handel, 2014; Pavlacky, Blakesley, White, Hanni, & Lukacs, 2012; Rota et al., 2009), and for WHWO provided opportunity for broadcasting calls followed by a period of listening (max 5 min total) before proceeding to the next point. To conserve time, surveyors immediately proceeded to the next point along a transect when white‐headed woodpecker was first detected. Thus, they strictly recorded detection–nondetection data, restricting the focus of monitoring to occupancy.

### 2.2 Population and sampling simulations

Following the initial 6‐year effort, we simulated occupancy‐based monitoring of white‐headed woodpeckers to inform potential future efforts. Recognizing the need for greater sampling effort to meaningfully quantify trends, however, we simulated surveys of ≥60 transects over 20 years.

Simulated populations experienced deterministic trends based on an exponential model,

(1) $$N_{t}=N_{1}\times \lambda_{N}^{t},$$

where *N*_*t*_ is population abundance in year *t* and λ_*N*_ is the proportion change in abundance per year (for theoretical basis, see Gotelli, 2001). We considered a range of trend scenarios of potential conservation concern, λ_*N*_ = {1.0, 0.98, 0.95, or 0.9}, that is, 0%, 33%, 64%, or 88% decline over 20 years. Simulated trends represented effect sizes for analyzing power. Positive trends (λ_*N*_ > 1.0) were less of a concern for informing prioritization of WHWO for conservation action and therefore not considered. Although real populations fluctuate stochastically, we lacked information for simulating specific levels of stochasticity, and deterministic trends provided clearer effect sizes for interpreting power estimates (see also Ellis et al., 2015; MacKenzie & Royle, 2005). We intended the range of simple deterministic trends considered here to inform surveillance monitoring aimed at documenting unanticipated change rather than particular ecological scenarios (Hutto & Belote, 2013).

We simulated population monitoring so as to explicitly represent the process of sampling discrete detection–nondetection data for continuously distributed populations (cf. Efford & Dawson, 2012; Ellis et al., 2014; *contra* MacKenzie & Royle, 2005). We conducted simulations using the rSPACE package (Ellis et al., 2014, 2015) in R (R Core Team, 2015). Simulations entailed (1) randomly distributing *N*_1_ home ranges across suitable habitat, (2) calculating the probability of encountering ≥1 white‐headed woodpecker at a survey point, (3) generating detection–nondetection data based on these encounter probabilities, and (4) randomly removing *N*_*t*_ × (1 − λ_*N*_) individuals from the landscape and repeating steps 2–3 for each remaining year *t *=* *2–20 (Appendix S1). We collected data for a region‐wide 300 m point grid, and later derived transect monitoring scenarios. Surveyors rarely recorded detections >150 m away (7% of 2011–2016 detections), so we simulated 150‐m fixed‐radius point surveys.

We consolidated point data to represent transect monitoring scenarios varying in sampling effort and allocation. A transect detection represented ≥1 detection at any given point along a transect on a given day. One home range (1‐km radius) could include multiple neighboring points, so point‐level detections within transects were spatially correlated, whereas ≥2 km transect spacing avoided spatial correlation in transect detections. Additionally, with transects, we were able to explore a fundamental issue in monitoring design: the relative merits of sampling intensively (e.g., more points per transect or repeat surveys) versus extensively (more transects).

We considered monitoring scenarios to accomplish two objectives: (1) identify levels of sampling effort capable of providing desirable power (≥80% chance to observe a decline given λ_*N*_ ≤ 0.98); (2) compare commonly considered sampling allocation strategies representing alternative targets of inference and spatial extents of sampling. We addressed objective 1 by varying sampling effort (*n*_transect_ ≥ 60; i.e., *n*_point‐surveys‐per‐year_ ≥ 1,200) with different trends and the historical sampling allocation of 10 points per transect surveyed twice every year. For objective 2, we focused on a long‐term decline scenario (λ_*N*_ = 0.98) and fixed sampling effort (*n*_point‐surveys‐per‐year_ = 1,200) while varying monitoring strategies. The historical allocation scheme represented an intended inference of relatively coarse‐scale trends. Alternative schemes included surveying shorter transects (8–3 points per transect), which targeted inference of finer‐scale trends by sampling smaller areas potentially occupied by fewer individuals. We also considered surveying transects only once per year, representing single‐survey occupancy approaches whose estimates provide temporal snapshots of populations, useful for inferring changes in abundance (Hutto, 2016; Latif et al., 2016). Finally, we considered surveying <100% of transects per year (i.e., panel designs; Bailey, Hines, Nichols, & MacKenzie, 2007; Urquhart & Kincaid, 1999) or repeating surveys at <100% of transects each year. Having fixed sampling effort, these alternate schemes allowed monitoring of more transects, which extended spatial sampling.

For simplicity, simulations assumed no false‐negative observer error (hereafter observer error), that is, white‐headed woodpeckers were always detected if present during a survey. Thus, detectability was determined exclusively by territorial movement between repeat surveys within a year. This assumption was defensible because call‐broadcast surveys reduce observer error and standardized surveys limit potentially confounding interannual variation in observer error (Mellen‐McLean et al., 2015). Additionally, we calibrated simulations with pilot data (Appendix S2). Encounter probabilities during a survey therefore reflected the number, location, size, and spacing of home ranges, informed by white‐headed woodpecker ecology; population size reflected calibration with pilot data and assumed trends (Appendices S1 and S2).

Spatial variability in detectability (i.e., encounter probabilities in simulations) emerged from variation in nesting habitat and random placement of home ranges within this habitat, which caused local abundance and proximity to centers of activity to vary among transects. Reflecting likely realities, detectability at occupied transects increased with increasing abundance and decreased with distance from home range centers (see Appendix S1). Analyses ignored this spatial heterogeneity, and thus informed study design while accounting for likely constraints on model complexity due to limited sampling effort. We initially considered smaller home ranges (600 m radius), but calibration to pilot data required compensatory adjustments to initial abundance, resulting in similar patterns in statistical power (Q. Latif, unpublished data). We restricted simulations to national forests, representing 77% (7.7 × 10^6^ ha) of potential habitat within the region (Figure 2).

### 2.3 Data analysis

**Figure 3:**
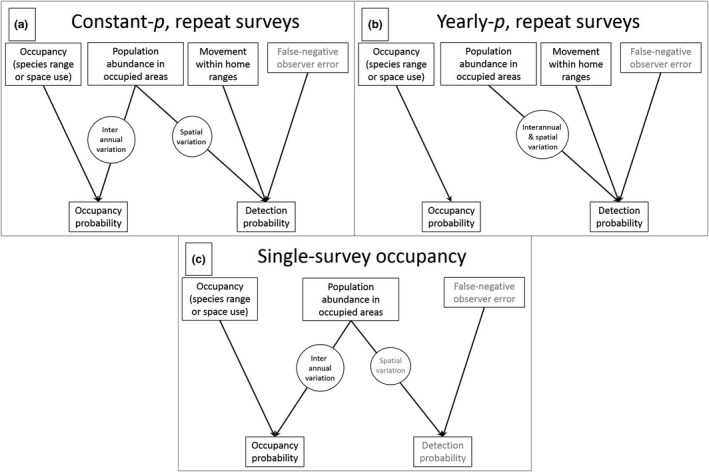
How model estimates reflect underlying processes under alternative monitoring approaches. Repeat‐survey occupancy estimates fundamentally quantify true occupancy (a, b) but can index abundance trends if detectability is held constant (b, *contra* A). Territorial movement influences repeat‐survey occupancy estimates (a, b), whereas single‐survey occupancy estimates represent population snapshots not influenced by movement (c). False‐negative observer error was not simulated but could influence estimates in reality

For scenarios yielding repeat‐survey data, we estimated trends with two different occupancy models representing commonly considered ways of correcting for detectability (*p*; e.g., Linden et al., 2017; Steenweg et al., 2016; Zielinski et al., 2013) to estimate occupancy probability (ψ; Figure 3a,b). One model allowed detectability estimates to vary interannually (*hereafter* the yearly‐*p* model; Figure 3a), whereas the other held detectability constant (*hereafter* the constant‐*p* model; Figure 3b; for model structures, see Appendix S3). Because individuals could move in or out of the surveyed area between repeat surveys within a year, these models quantified the probability of a transect intersecting ≥1 home range, hereafter *true occupancy*, which describes species range or space use (Efford & Dawson, 2012; MacKenzie & Royle, 2005). The yearly‐*p* model allows detectability to change with changing abundance (Royle & Nichols, 2003) to better estimate true occupancy. The constant‐*p* model misspecifies true occupancy, but is frequently considered and may be selected for parsimony in applied studies (e.g., Zielinski et al., 2013). Additionally, having controlled for observer error (e.g., if nonexistent as in simulations, or controlled via standardized surveys), the constant‐*p* model coerces occupancy estimates to reflect any interannual changes, shifting the target of inference to abundance (Figure 3b).

For scenarios yielding single‐survey data, we estimated trends using logistic regression (see structure in Appendix S3). Having excluded observer error in simulations, logistic regression models estimated probability of ≥1 individual's physical presence during a survey, hereafter *probability of physical presence*. Single‐survey scenarios represented single‐survey occupancy approaches, in which replicate surveys occur within a narrow enough timeframe for detectability to quantify observer error so that occupancy estimates quantify probability of physical presence (e.g., double‐observer and removal designs; Nichols et al., 2008; Rota et al., 2009). By omitting observer error from simulations, however, replicate surveys were unnecessary to quantify physical presence. Probability of physical presence represents a temporal snapshot of a population unaffected by territorial movement expected to closely track abundance (Figure 3c; Hutto, 2016; Latif et al., 2016). Additionally, surveying transects only once allowed us to monitor twice as many transects, increasing sampling extent. In practice, auxiliary sampling (e.g., recording detection timing or deploying multiple observers) would account for observer error (Nichols et al., 2008; Rota et al., 2009), likely adding to uncertainty in trend estimates. Ignoring observer error in both single‐ and repeat‐survey scenarios, however, made their comparison informative.

We quantified occupancy trends as proportion yearly change in odds occupancy, $\lambda_{\psi }=\frac{\psi_{t+1}/\left(1-\psi_{t+1}\right)}{\psi_{t}/\left(1-\psi_{t}\right)}$ (MacKenzie et al., 2006). We analyzed detection–nondetection data with fixed year effects and subsequently calculated least‐squares trends in yearly occupancy estimates (see also Ellis et al., 2014). We quantified statistical power as percent simulations when 95% Bayesian credible intervals (BCIs) for the estimated trend for the study period ($\hat{\lambda_{\psi }}$ where $\text{logit}\left(\psi_{t}\right)=\beta_{0}+\log\left(\lambda_{\psi }\right)\times t$; p. 200, MacKenzie et al., 2006) fell below 1. We also calculated root mean squared error for trend estimates (${\text{RMSE}}_{N}=\sqrt{\text{mean}(\hat{\lambda_{\psi }}-\lambda_{N})}$, ${\text{RMSE}}_{\psi }=\sqrt{\text{mean}(\hat{\lambda_{\psi }}-\lambda_{\psi })}$). When quantifying true occupancy, we considered occupied transects to be those with encounter *p* ≥ .05. Having found extremely limited statistical power with yearly‐*p* estimates (see Objective 1 Results), we primarily assessed sampling allocations (Objective 2) for constant‐*p* and logistic regression models, but then tested yearly‐*p* again with better allocation. Furthermore, given likely targets of inference, we considered RMSE_*N*_ most relevant to constant‐*p* and logistic regression models, and RMSE_ψ_ relevant to the yearly‐*p* model. For additional methods and rationale, see Appendix S3.

## 3 RESULTS

### 3.1 Occupancy, abundance, and estimator behavior

**Figure 4:**
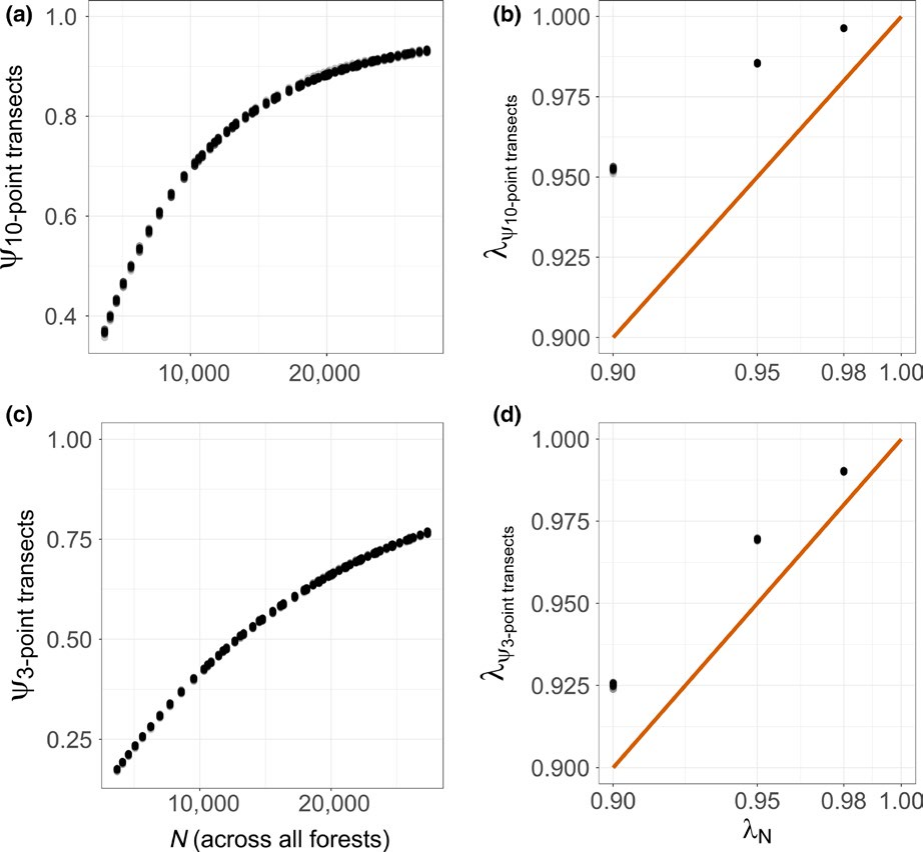
True occupancy (ψ) versus abundance (*N* = number of individuals across all 7,676,971 ha of potential habitat in Oregon and Washington national forests; a, c) and correspondence of (odds) occupancy (λ_ψ_) with abundance trends (λ_*N*_; b, d) for simulated white‐headed woodpecker populations. Thirty replicate populations monitored for 20 years for each trend scenario are depicted when surveyed at transects consisting of 10 points (a, b) or three points (c, d) each. In panels b and d, the red line indicates 1:1 correspondence (desirable for inference) between occupancy and abundance trends

Comparing true occupancy (proportion transects with encounter *p* ≥ .05) and abundance informed understanding of statistical power and estimator properties. True occupancy related positively with abundance but plateaued at higher abundances (Figure 4a,c). True occupancy declines lagged abundance declines (Figure 4b,d). With shorter transects, true occupancy corresponded better but still imperfectly with abundance (Figure 4c,d).

**Figure 5:**
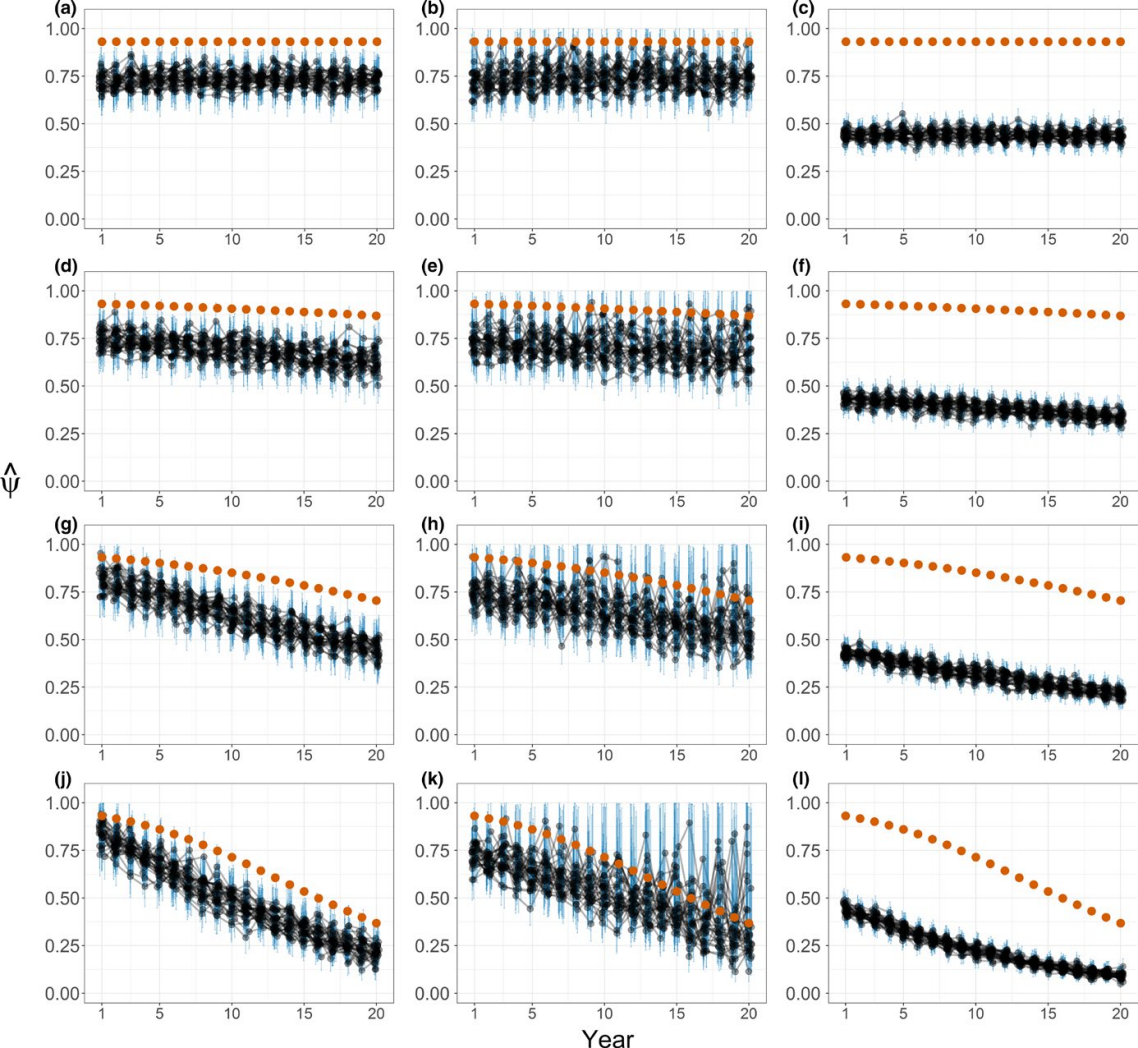
Yearly occupancy estimates from simulated regional white‐headed woodpecker monitoring. Simulated trends were λ_*N*_ = 1 (a–c), 0.98 (d–f), 0.95 (g–i), and 0.9 (j–l). Repeat‐survey occupancy estimates assumed constant detectability (a, d, g, and j) or variable detectability among years (b, e, h, and k). Single‐survey estimates assumed perfect detectability (c, f, i, and l). Thirty simulations of monitoring transects of 10 points each for 20 years are represented for each scenario (*n *=* *150 and 300 transects for repeat‐ and single‐survey scenarios, respectively). Black dots and blue vertical bars show yearly estimates and 95% BCIs jittered for display. Black lines connect estimates from consecutive years for individual simulations. Red dots show mean true occupancy for 30 simulations, that is, proportion of all possible transects occupied

**Figure 6:**
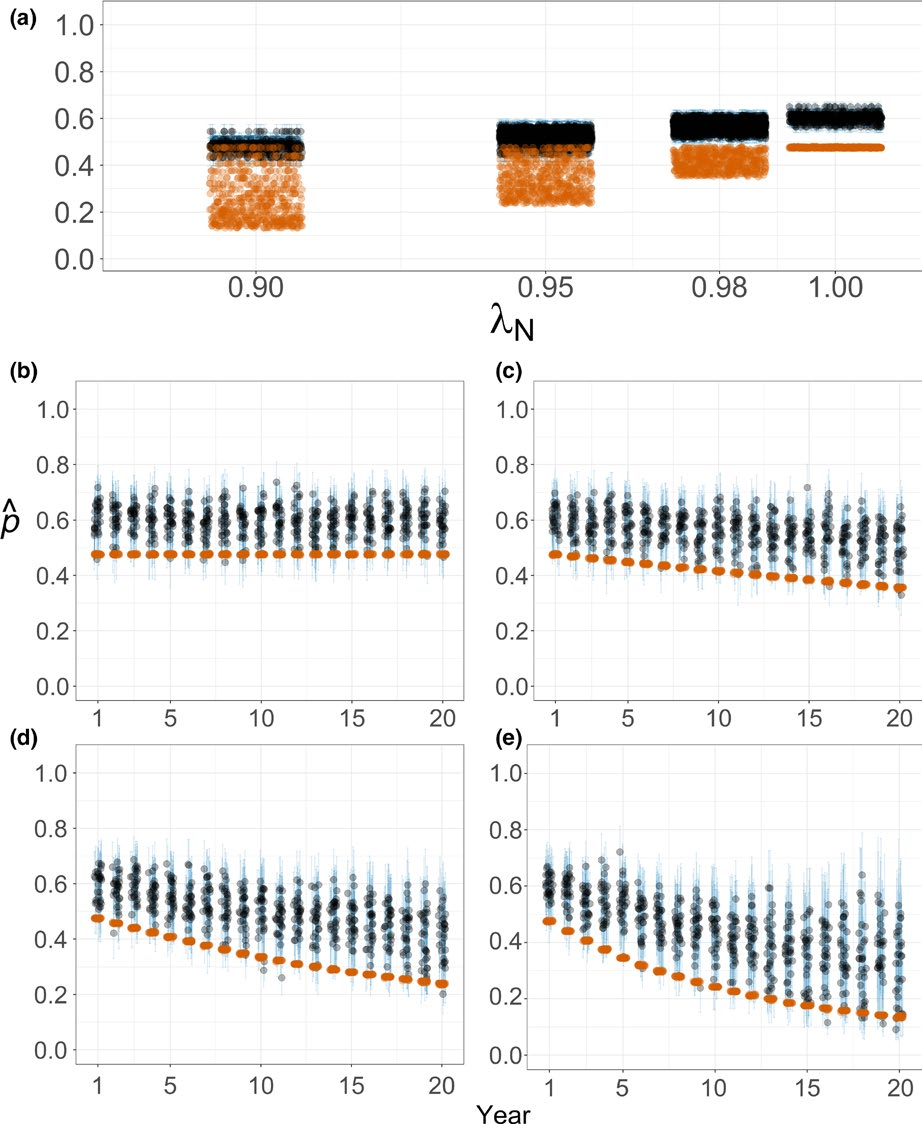
Detection probability (*p*; black = median, blue = 95% BCIs) estimates from repeat‐survey occupancy models and encounter probabilities (red; i.e., true detectability) for simulated white‐headed Woodpecker regional monitoring. Scenarios entailed monitoring 150 transects of 10 points each surveyed twice yearly for 20 years. Estimates assume constant detectability (a) or variable detectability among years (b–e; jittered horizontally for display). Encounter probabilities are median values for occupied transects (i.e., with encounter *p* ≥ .05). Simulated trends were λ_*N*_ = 1.0 (a, b), 0.98 (a, c), 0.95 (a, d), or 0.9 (a, e) (*n *=* *30 simulations per scenario)

Occupancy estimates remained constant with no abundance trend and declined with declining abundance (Figure 5). Detectability estimates declined with declining abundance either across scenarios (constant‐*p* estimates) or through time (yearly‐*p* estimates; Figure 6). Yearly‐*p* estimator precision was less than for constant‐*p* estimates and declined with declining abundance (Figures 5 and 6).

Detectability estimates followed the behavior of encounter probabilities at occupied transects but were generally higher, that is, positively biased (Figure 6), making occupancy estimates negatively biased (Figure 5). Logistic regression estimates deviated even more from true occupancy (Figure 5c,f,i,l), reflecting the differing target of inference (i.e., probability of physical presence; see Section 2 and Appendix S3).

### 3.2 Objective 1: Sampling effort

**Figure 7:**
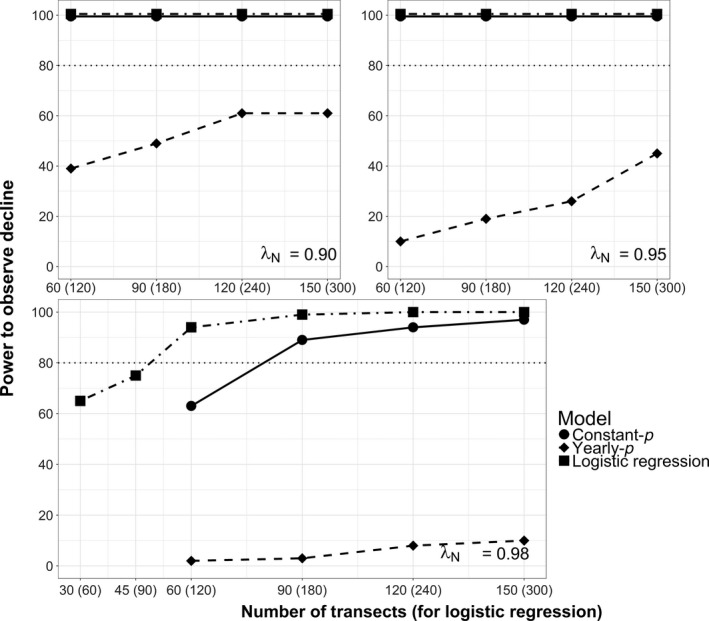
Simulation‐based power to observe white‐headed woodpecker regional occupancy trends (percent simulations with 95% BCI <1). Scenarios varied in number of transects, monitoring approach (constant‐*p* or yearly‐*p* occupancy models, or logistic regression), and trend (λ_*N*_ = exponential change in abundance). For all scenarios, transects consisted of 10 survey points surveyed twice (occupancy models) or once (logistic regression) per year. Constant‐*p* assumed constant detectability, whereas yearly‐*p* allowed variable detectability among years. Logistic regression allowed double the number of transects by analyzing single‐survey data

With historical survey allocation, statistical power increased with increasing sampling effort and stronger population declines (Figure 7). The constant‐*p* model and logistic regression (with single‐survey allocation) generally provided adequate power (≥80% chance of observing a decline). Power was only inadequate with a small effect size (λ_*N*_ = 0.98) and minimal sampling effort (*j *≤* *60 and 90 transects for constant‐*p* and logistic regression, respectively). In contrast, power was never adequate with the yearly‐*p* model. Spurious trends were rarely observed (1.5% of simulations in which λ_*N*_ = 1).

**Figure 8:**
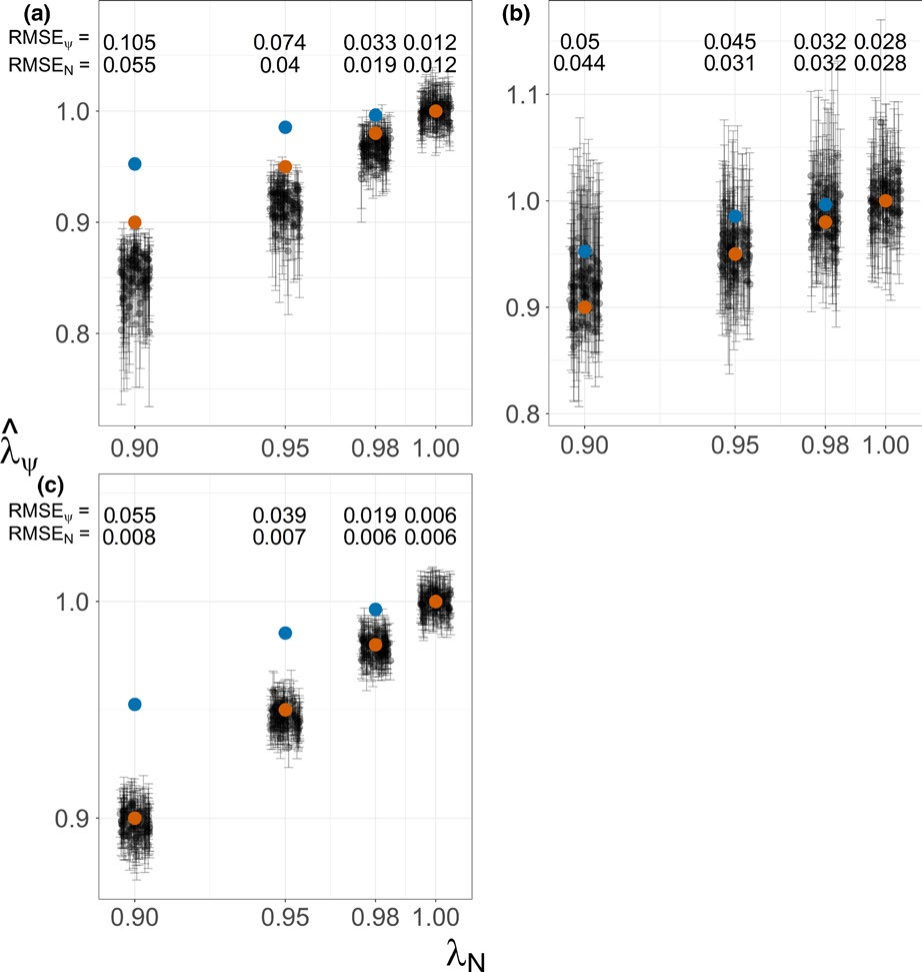
Correspondence of estimated occupancy trends ($\hat{\lambda_{\psi }}$) with true abundance (λ_N_) and occupancy (λ_ψ_) trends. Trends were estimated with repeat‐survey constant‐*p* (a) and yearly‐*p* (b) occupancy models, and logistic regression analyzing single‐survey data (c). Red and blue dots mark perfect correspondence with actual abundance and occupancy trends, respectively. Root mean squared error quantifies the overall estimation error with respect to abundance (RMSE_N_) and occupancy (RMSE_ψ_) trends

With no abundance or occupancy trends, models estimated actual trends with minimal error and no apparent bias, but error and bias grew with increasing trend (Figure 8). The constant‐*p* model increasingly overestimated declines with steeper abundance declines, although abundance trends were estimated better (RMSE_*N*_ ≤ 0.055) than occupancy trends (RMSE_ψ_ ≤ 0.105). Yearly‐*p* trend estimates were centered between actual abundance and occupancy trends (RMSE ≤ 0.05). Models fitted to single‐survey data estimated true abundance trends with the least error (RMSE_*N*_ ≤ 0.008) and no obvious bias.

### 3.3 Objective 2: Sampling allocation

**Figure 9:**
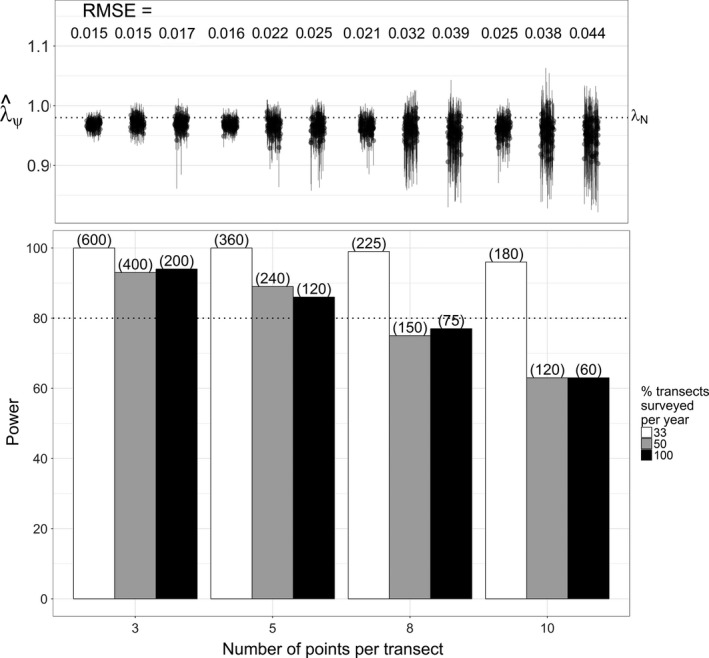
Statistical power (percent simulations with 95% BCI <1) and trend estimation error (RMSE) for the repeat‐survey constant‐*p* occupancy model under alternative sampling allocation strategies. Error is calculated relative to the actual abundance trend, λ_*N*_ = 0.98 (RMSE = RMSE_*N*_). Strategies depicted involve monitoring rotating subsets of transects each year (bar color) or fewer points per transect (*x*‐axis) in exchange for monitoring more transects. Parenthetic values indicate the total number of transects monitored over the 20‐year study period

**Figure 10:**
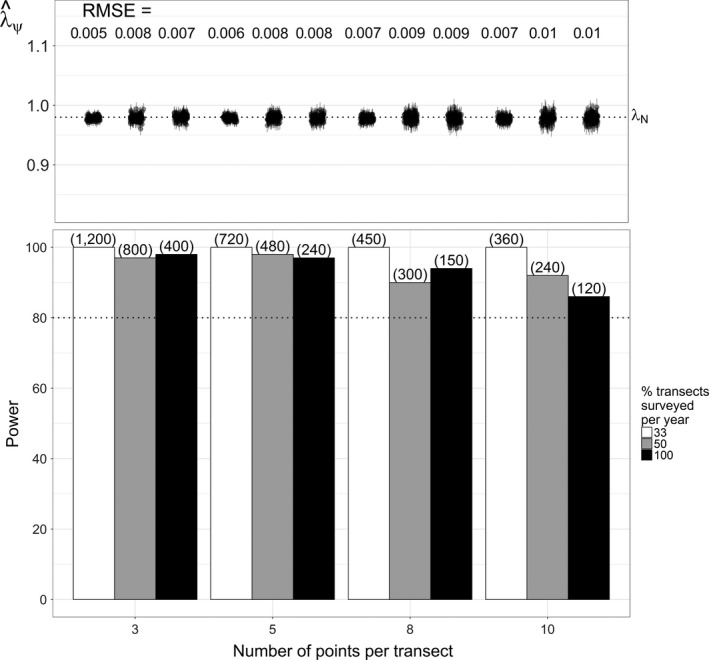
Statistical power (percent simulations with 95% BCI <1) and trend estimation error (RMSE) for the single‐survey logistic regression under alternative sampling allocation strategies. Error is calculated relative to the actual abundance trend, λ_*N*_ = 0.98 (RMSE = RMSE_*N*_). Strategies depicted involve monitoring a rotating subsets of transects each year (bar color) or fewer points per transect (*x*‐axis) in exchange for monitoring more transects. Parenthetic values indicate the total number of transects monitored over the 20‐year study period

Monitoring strategies that targeted inference of finer‐scale trends in space use or abundance and extended sampling spatially generally provided more power and less estimation error than the historical strategy. Power improved and estimation error decreased when monitoring shorter but more transects (Figures 9 and 10). Power was greatest and error (RMSE_*N*_) least when monitoring the probability of physical presence with single‐survey data (Figures 8c and 10). In contrast, we found the least power and greatest error (RMSE_ψ_) when attempting to monitor true occupancy with repeat surveys and the yearly‐*p* model (Figure 8b). Interestingly, despite more explicitly targeting inference of occupancy, yearly‐*p* trends estimates did not estimate occupancy trends with any less error (RMSE_ψ_ ≤ 0.105) than logistic regression (RMSE_ψ_ ≤ 0.055). Although it provided adequate power in many scenarios, the constant‐*p* model tended to overestimate both occupancy and abundance declines (Figures 8a and 9).

**Figure 11:**
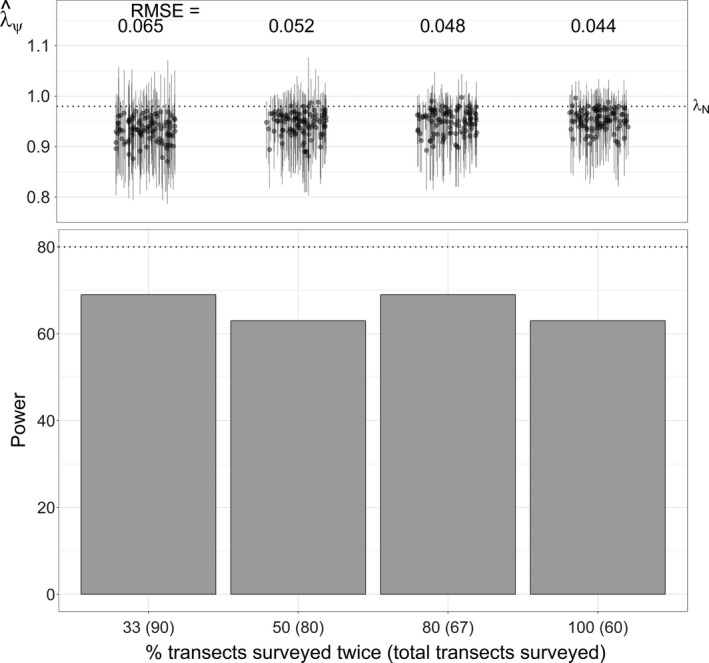
Statistical power (percent simulations with 95% BCI <1) and trend estimation error (RMSE) for the repeat‐survey constant‐*p* model for scenarios that vary the proportion of transects surveyed a second time each year in exchange for monitoring more transects. Error is calculated relative to the actual abundance trend, λ_*N*_ = 0.98 (RMSE = RMSE_*N*_). Parenthetic values indicate the total number of transects monitored over the 20‐year study period

Panel designs with relatively small panels (33% of transects surveyed each year) also improved power and reduced error, although larger panels (50% of transects surveyed each year) did not provide notable gains. Conducting fewer repeat surveys in exchange for more transects also did not substantively affect power and tended to increase estimation error (Figure 11).

The design that maximized power and minimized error with constant‐*p* and logistic regression models was a 33% panel design with 3 points per transects. Even with this design, the yearly‐*p* model provided inadequate power (13%), although trend estimation error was less than with the historical design (RMSE_ψ_ = 0.009; *n*_sim_ = 100; *n*_transect_ = 600 over 20 years; compare with Figure 7b).

## 4 DISCUSSION

Our simulations suggested minimum levels of sampling effort needed to provide adequate power while also informing study design for monitoring WHWO with explicit targets of inference. With the historical design of surveying transects with 10 points each twice a year to target coarse‐scale trends in true occupancy (species range or space use), we found 60–90 transects could be sufficient for desirable power. This design would require holding detectability constant across years, however, which would force occupancy estimates to index abundance (constant‐*p* model) and cloud potential inference. Surveying shorter transects (i.e., closer to the span of one home range) using a 33% panel design could allow stronger and clearer inference of abundance trends, extend sampling spatially, and improve power. For further improvements to power and inference, we could survey transects only once per year to monitor the probability of physical presence while accounting for observer error. In contrast, sampling designed to document changes in true occupancy did not appear feasible at sampling levels considered here.

### 4.1 Sampling resolution and scale of inference

Our results further emphasize the benefits of sampling at resolutions (i.e., unit size, grid cell size) approximating the size of an individual home range documented by others (Efford & Dawson, 2012; Linden et al., 2017). Finer resolution sampling generates occupancy estimates that more closely track abundance. This estimator property should be desirable for practitioners who monitor occupancy in lieu of abundance primarily on pragmatic grounds.

Single‐survey sampling can similarly benefit monitoring of territorial animals by providing temporal snapshots of populations unaffected by movement and therefore closely related to abundance (Hutto, 2016; Latif et al., 2016). We simulated an ideal world with no observer error wherein single‐survey estimates were readily interpretable as the probability of physical presence. In reality, some observer error is likely, requiring auxiliary sampling to estimate a snapshot probability of physical presence. Auxiliary measurements of detection timing or covariates of observer error could inform bias correction with minimal additional survey effort (Lele, Moreno, & Bayne, 2012; Rota et al., 2009). For monitoring white‐headed woodpeckers, analysis of detection timings recorded historically (Mellen‐McLean et al., 2015) combined with published guidelines (MacKenzie & Royle, 2005) could inform optimal survey length for single surveys. Other approaches are described but would require more effort or are designed to inform abundance rather than occupancy estimates (e.g., multiple observers, replicated camera or track stations, distance sampling; Amundson et al., 2014; Nichols et al., 2008). Lacking data on observer error, naïve occupancy could usefully index abundance if we are confident that observer error does not vary interannually and therefore cannot confound trend estimation (e.g., with standardizing bird surveys; Hutto, 2016).

Observer error can vary with local abundance (Royle & Nichols, 2003), potentially introducing noise not represented in our simulations. Larger sampling units potentially occupied by multiple individuals would be most prone to such variability, so aligning sampling resolution with home range size would be desirable even with a single‐survey design.

A single‐survey design would require consistently conducting surveys when individuals are readily detectable. With sensitivity of nest survival to temperature (Hollenbeck et al., 2011), climate change may alter nesting phenology, potentially influencing responsiveness to call broadcasts. Such changes could necessitate adjusting the timing of surveys, which could be informed by targeted repeat surveys, as are commonly implemented for birds (Latif, Fleming, Barrows, & Rotenberry, 2012; Rota et al., 2009).

Our results indicate challenges for monitoring to infer changes in species range (coarse‐scale) or space use (finer scale) as in yearly‐*p* scenarios here. By definition, occupancy only declines when abundance declines enough to result in local extirpation, so true occupancy declines could indicate strong need for conservation. More intensive sampling in areas or years with low abundance, however, may be needed to correctly identify occupancy declines. At finer scales, spatial heterogeneity in detectability arising from variability in local abundance and home ranges that lack definitive boundaries can limit accurate estimation of space use (Efford & Dawson, 2012). Biases in occupancy and detectability estimates observed here likely primarily reflect these effects. Including habitat relationships with occupancy in analytical models (omitted from simulations) might help by accounting somewhat for spatial heterogeneity in the data, but effects on detectability of varying local abundance and proximity to home ranges at occupied transects would remain. Effectively estimating species distribution at any scale may require substantial spatial or temporal replication within sampling units (Pavlacky et al., 2012; Valente et al., 2017). Given likely demands on funding, such approaches may be feasibly implemented only infrequently (e.g., Cruickshank, Ozgul, Zumbach, & Schmidt, 2016). Alternatively, predictive models (e.g., Hollenbeck et al., 2011; Latif et al., 2015; Wightman, Saab, Forristal, Mellen‐McLean, & Markus, 2010) could supplement trend monitoring by identifying changes in habitat.

Nested surveys (e.g., points along transects) can inform hierarchically structured models capable of estimating patterns or trends at multiple scales (Pavlacky et al., 2012; Rota et al., 2009; Royle & Kéry, 2007). Multiscale inference would require sufficient sampling at all scales of interest, however, which may be beyond resources available for many monitoring programs (Valente et al., 2017). Our initial attempts found inadequate sampling for meaningful multiscale inference (Q. Latif, unpublished data), so we abandoned such approaches here.

### 4.2 Sampling extent

Spatially extensive sampling is theoretically advantageous when monitoring spatially heterogeneous populations (Rhodes & Jonzén, 2011). In our simulations, spatial heterogeneity emerged from uneven distribution of habitat and random variation in local abundance among occupied transects. The benefits observed here with shorter transects and single surveys could reflect advantages of spatially extended sampling. Panel designs, however, did not inherently change the target of inference, and so their results more definitively demonstrated potential advantages with spatially extended sampling. In contrast, ignoring heterogeneity inherent in continuously distributed territorial species may obscure advantages of panel designs (Bailey et al., 2007; Urquhart & Kincaid, 1999).

Not all spatial extensions to sampling were beneficial. Given a repeat‐survey design, we gained nothing by reducing repeat surveys to monitor more transects. Such strategies require high detectability (MacKenzie & Royle, 2005) likely uncharacteristic of sparsely and continuously distributed territorial species (see above). The lack of benefit with 50% panels may reflect site fidelity, fixed at 100% in our simulations. By monitoring different transects in successive years, the number and distribution of individuals along surveyed transects varied interannually, potentially obscuring trends. White‐headed woodpecker do exhibit site fidelity (Garrett et al., 1996), so panel design benefits could trade‐off with benefits of sampling the same sets of individuals in successive years. In reality, however, population processes (e.g., dispersal and turnover) could also obscure trends. The extent of paneling needed to benefit power would therefore depend on levels of spatial versus temporal heterogeneity in population trends (Rhodes & Jonzén, 2011), the latter of which was omitted from simulations here. Additionally, panel designs could limit study of processes underlying occupancy dynamics, for example, colonization and persistence (Bailey et al., 2007).

### 4.3 Study limitations

Our simulations did not include spatial or temporal stochasticity in population dynamics, individual movement between years, or behavioral interactions between neighbors all of which could modulate occupancy estimates or trends (Reynolds, Wiens, Joy, & Salafsky, 2005; Sauer, Fallon, & Johnson, 2003; Warren, Veech, Weckerly, O'Donnell, & Ott, 2013). By not accounting for these realities, power estimates may be liberal, and therefore probably best used to inform a lower bound for sample size. Accordingly, we recommend ≥120 or ≥90 transects with single‐survey or repeat‐survey monitoring, respectively, of white‐headed woodpeckers across our study region.

Our treatment of site fidelity, however, is conservative. For simplicity, we simulated populations with 100% site fidelity and zero immigration or recruitment, and to avoid artifacts of these assumptions, analysis models assumed occupancy varied independently among years. In reality, models correctly specifying uncertainty arising from additional population processes (e.g., Royle & Kéry, 2007) could improve power to observe trends (although with likely increased data demands). Models that correctly specify habitat relationships with occupancy could also help. Given the potentially counteracting features of simulations, we expect power estimates were sufficiently informative to compare alternative study designs.

Simulations ignored spatial variation in home range size, which can confound interpretation of occupancy estimates and trends drawn from repeat surveys (Efford & Dawson, 2012). Simulations including such realities could further inform repeat‐survey monitoring. Alternatively, single‐survey monitoring would avoid this issue, and could be complemented with focused study of space use dynamics.

Our treatment of survey cost did not fully account for travel time among transects. We expect little difference in cost of repeating a survey versus surveying a new transect, but travel time could limit transect number more than length. To fully inform study design, biologists would need to attach costs to scenarios explored here. For white‐headed woodpeckers, clustering transects with sufficient spacing for statistical independence (e.g., 2–5 km assuming home ranges ≤1 km radius) could reduce travel time, although potentially raising the need to account for spatial heterogeneity at coarser scales (e.g., among sub‐regions).

### 4.4 Additional considerations and broader implications

Agency biologists often conduct repeat surveys to estimate detectability and thereby improve credibility of trend estimates. This strategy potentially implies an overly rigid allocation of effort between sampling to inform occupancy versus detectability. Repeat surveys of mobile species may unwittingly focus effort toward tracking distributional shifts, which can be harder to observe and not necessarily more relevant to conservation than changes in abundance. Additionally, practitioners often discount the potential for detectability to change with changing abundance (e.g., Ahumada, Hurtado, & Lizcano, 2013; van Strien, van Swaay, & Termaat, 2013; Zielinski et al., 2013), which may limit explicit inference of abundance trends versus range dynamics from occupancy‐based trend estimates. Estimating detectability is only useful if doing so improves inference of underlying population processes or accounts for interannual variability in observer error. The former requires considering which processes can be more readily inferred by accounting for detectability at the scale it is measured. If instead biologists are solely concerned with controlling observer error, monitoring of population indices may be more cost‐effective while providing equivalent or stronger inference of population change (Hutto, 2016; Johnson, 2008; Welsh et al., 2013).

Despite growing sophistication of occupancy models (Bailey, MacKenzie, & Nichols, 2014), heterogeneity arising from locally varying abundance and poorly defined home range boundaries (Efford & Dawson, 2012) will continue to challenge monitoring efforts, especially where funding constrains data and, consequently, model complexity. Simulations can help explore our capacity for inference with models necessarily misspecified due to limited data. General power formulas available for occupancy models ignore spatial heterogeneity (Guillera‐Arroita & Lahoz‐Monfort, 2012; MacKenzie & Royle, 2005). Spatially explicit simulations therefore complement these tools for tailoring sampling designs to particular study systems.

Information on regional trends should be combined with information on various population parameters measured at different scales to fully inform species conservation status (Nichols & Williams, 2006). For example, other studies currently underway examine forest management effects on white‐headed woodpecker nest densities, nest survival, and habitat use (Mellen‐McLean et al., 2015). Statistical models can now integrate multiple sources of data to better inform parameter estimation (Dorazio, 2014; Nichols et al., 2008). Simulations that explicitly and distinctly describe population from observation processes could inform sampling design to support these approaches.

## CONFLICT OF INTEREST

None declared.

## AUTHOR CONTRIBUTIONS

Q. Latif implemented simulations and data analysis, and drafted the manuscript. M. Ellis, V. Saab, and K. Mellen‐McLean provided substantive feedback during conceptual framing, simulation design and implementation, and manuscript preparation.

## DATA ACCESSIBILITY

R scripts for initiating simulations using the rSPACE package and BUGS code defining data analysis models are provided online supporting information.
