# Supplementary material for: Simulations inform design of regional occupancy‐based monitoring for a sparsely distributed, territorial species
Source: Ecol Evol. 2017 Dec 20;8(2):1171–85. doi: 10.1002/ece3.3725 (PMC5773320; doi:10.1002/ece3.3725)
Supplement: Supplementary file 1 [file ECE3-8-1171-s001.docx]

Appendix A. Population simulations and detection data generation

We used the R package, rSPACE (Ellis et al. 2014, Ellis et al. 2015), to conduct population simulations and generate point-level detection data within each national forest. Point-level data were subsequently re-sampled and consolidated into regional transect detection data. For each proposed abundance trend, we simulated and sampled 30 populations, resulting in 30 point-level detection datasets. We then sampled with replacement from these to generate transect-level datasets consolidated across forests. We resampled and subset point-level data to compile transect datasets, and we further subset transect data as needed for each sampling scenario. Generating transect data by sampling with replacement from point-level data reduced computation time and was defensible because a transect sample for any given scenario represented data for only a small subset of potential survey points (max = 6000 of 439,655 points). The steps followed and criteria used to complete this process are outlined here:

1. *Map suitable nesting habitat* – We defined suitable nesting habitat using a published habitat suitability index (HSI) model developed for unburned forests (Latif et al. 2015), whereby sites (30m pixels) with HSIs > 0.36 were considered suitable for nesting (Figure A1). The resulting habitat suitability map constrained the location of home range centers but not movement within home ranges.
2. *Locate potential survey points* – We generated a 300m point grid within potential habitat (any 30m pixel with ≥5% large-cone pine-dominated (or co-dominated; *Pinus ponderosa*, *P. jeffreyi*, *P. lambertiana*, *P. monticola*) forest within a 150 m radius neighborhood; tree species dominance classified by GNN; Ohmann and Gregory 2002). We retained points from this grid capable of forming ≥ 1 transect (a continuous line of 10 points oriented north-south or east-west) as potential survey points.
3. *Locate home range centers* – We located home range centers for simulated populations according to 3 criteria:
   1. A home range center could only occur within suitable nesting habitat as defined above.
   2. The minimum distance between neighboring home range centers was 300m, reflecting the minimum inter-nest distances observed at our study area in the East Cascade Mountains (i.e., data analyzed by Latif et al. 2015).
   3. The total number home range centers in monitoring year 1 was fixed so that overall density across each National Forest was 0.2522 home ranges per 70 ha. We determined this value from preliminary simulations that calibrated simulated detection data against pilot data (Appendix B). In monitoring years 2–20, abundance was determined by applying the assumed population trend (e.g., λ*_N_* = 1.0, 0.98, 0.95, or 0.9) to initial abundance. Within the Gifford Pinchot NF, availability of suitable nesting habitat limited initial abundance slightly below the prescribed 0.2522 per 70 ha level. Thus, initial abundance varied slightly among simulations (mean *N*_1_ = 27,340, range = 27,316 – 27,364, *n* = 120 simulated populations).

An example set of potential survey points and home range centers for an area of the Deschutes NF is shown in Figure A1.


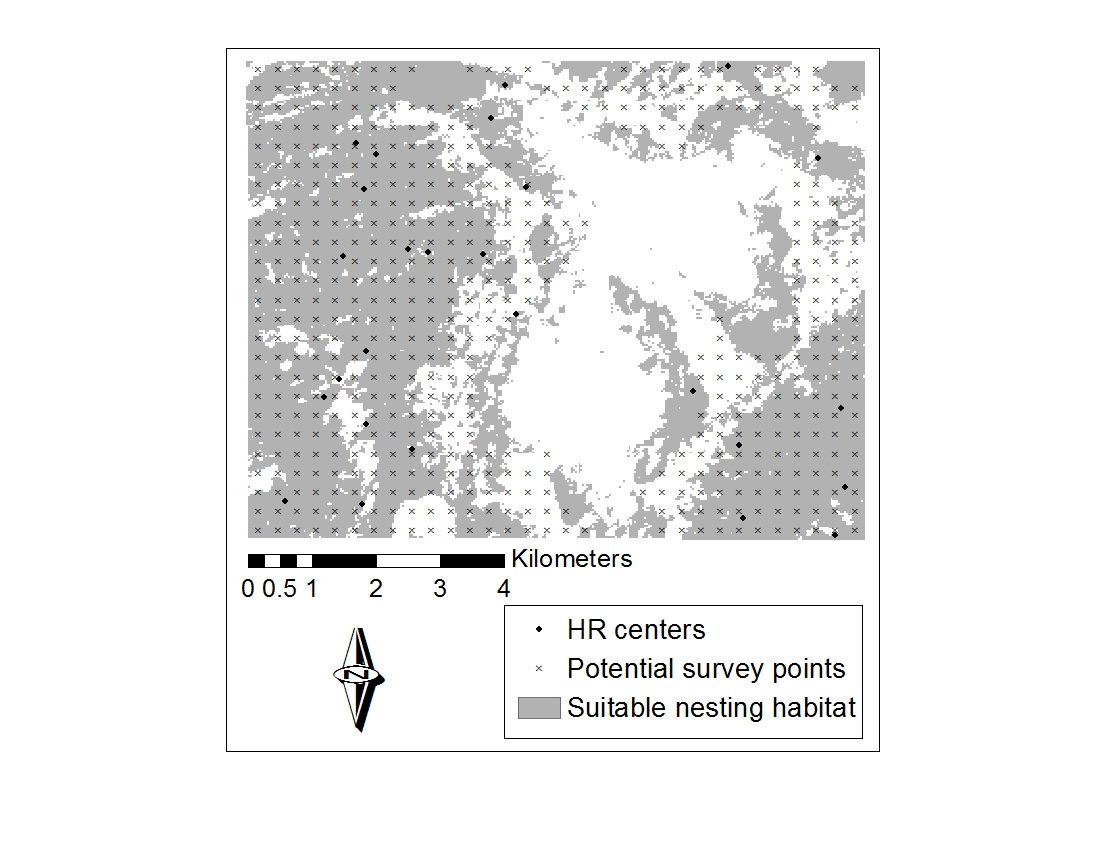


Figure A1. Map of simulated home range centers in monitoring year 1, potential survey points, and suitable nesting habitat within a portion of the Deschutes NF. Home range centers (but not movement within home ranges) are constrained to occur within suitable nesting habitat (defined by a published model) and spaced 300 m minimum from neighboring home range centers. Potential survey points form a 300 m grid generated within potential habitat defined by coverage of pine-dominated forest (not shown).

1. *Quantify space use and encounter probabilities within home ranges* – We assumed that the location of an individual within its home range at any given time was determined by a bivariate normal distribution (variables – UTM easting and northing) with a mean set to the home range center and a variance so that each individual would occur within 1 km of the home range center with probability = 0.95. White-headed woodpeckers forage in various habitats and the predictive model describing suitable nesting habitat was informed by territory-level attributes relevant to foraging (Hollenbeck et al. 2011, Latif et al. 2015). We therefore imposed habitat restrictions on the location of home range centers but not on space use within the home range.
2. *Point-specific encounter probabilities* – We aggregated across home ranges to calculate an encounter probability for each 30m pixel *i*: $p_{i}= 1-\prod_{h=1}^{max(h)} \left( 1-p_{h} \right)^{2}$, where *p_h_* is the probability of encountering each member of a breeding pair associated with a single home range (Figure A3B). For each home range, the quantity 1 – *p_h_* was squared to reflect the presence of 2 individuals per home range. For each survey point *j*, we calculated the encounter probability: $p_{j}= 1-\prod_{i}^{I} \left( 1-p_{i} \right)$, where *I* = the number of pixels within 150m of point *j* (Figure A3C).


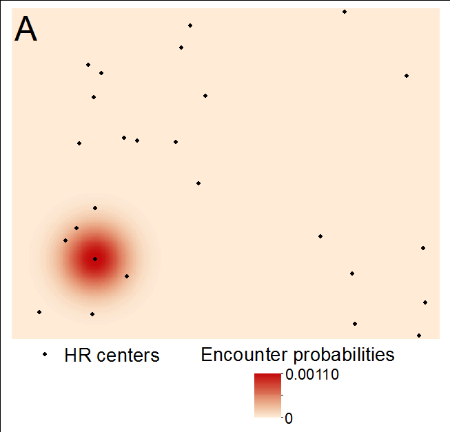

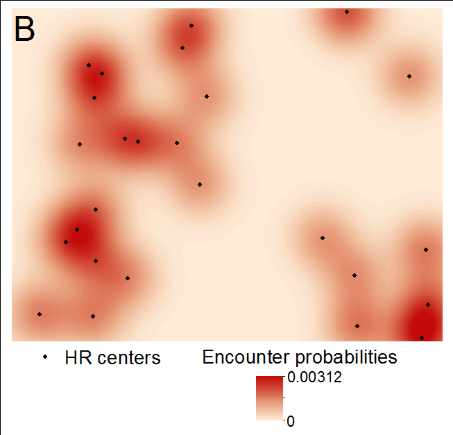

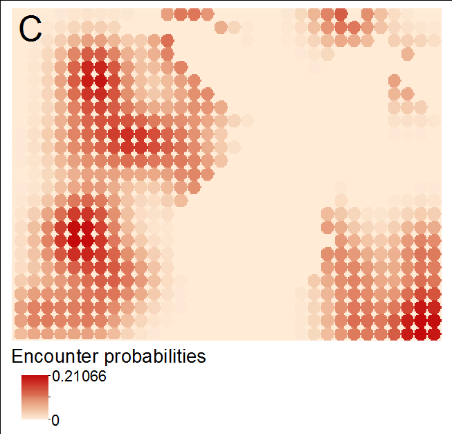


Figure A3. Steps in calculating point-specific encounter probabilities. We first generate encounter probabilities specific to each home range (A), then aggregated across home ranges (B), and finally aggregated across pixels within 150 m of each survey point to generate an encounter probability for each survey point *j* (C).

1. *Point detection data –* We simulated two survey occasions for each potential survey point for each of 20 years. For each occasion, WHWO detection at point *j* was determined by a Bernoulli distribution with success probability = *p_j_* calculated in Step 5.
2. *Identify transects* – A transect consisted of a continuous line of potential survey points with 300 m spacing. We identified enough transects such that all potential survey points were assigned to ≥ 1 transect.
3. *Transect sets* – We identified a series of transect sets such that points along member transects within any set were spaced ≥ 2 km from points along any other member transect, and all transects were assigned to ≥ 1 set.
4. *Transect detection data and encounter probabilities* – For each iteration of a sampling scenario, we drew the requisite sample of transects from a randomly selected transect set (see Step 8). We weighted transect samples across forests by the total number of transects identified in each forest, which was constrained by availability of potential habitat (i.e., transect sets in forests with more potential habitat were more heavily sampled). For each transect *k* in year *t*, we considered WHWO detected if detected ≥ 1 point 1 – *J*, where *J* ϵ {3,5,8,10}, along transect *k*. For purposes of calculating true occupancy, the encounter probability for transect *k* as $p_{k}= 1-\prod_{j}^{J} \prod_{i}^{I} \left( 1-p_{ij} \right)$, whereby transect *k* was considered occupied if *p_k_* ≥ 0.05 (Table A1).
5. *Transect data subsetting* – We further subset transect detection data as needed for scenarios representing alternative sampling allocation schemes. For panel design scenarios, we divided transects into equal-size panels and retained data for one panel in each year on a rotating basis. For scenarios with < 10 points per transect, we aggregated across a subset of points along each transect (see Step 9). For reduced repeat-visit scenarios, we randomly selected the requisite portion of transects (33%, 50%, or 80%) for each year and retained data from the second survey for those transects in that year (i.e., second-survey data were eliminated for a different subset of transects in each year). All transects represented in any dataset were spaced ≥ 2 km apart and thus sampled different sets of individuals.

Table A1. Example calculation of transect detection data (*y_k_*) and encounter probabilities (*p_k_*) from respective point-level values (*y_j_*, *p_j_*). *y_k_* = 1 if any *y_j_* = 1, where *J* = 3 points per example transect. Transect encounter probabilities are aggregated across points: $p_{k}= 1-\prod_{j}^{J} \left( 1-p_{j} \right)$. Both example transects were occupied (*p_transect_* ≥ 0.05) even though WHWO were detected at only one.

| Example transect | Quantity | Point values (*y_j_*, *p_j_*) | | | Transect values (*y_k_*, *p_k_*) |
| --- | --- | --- | --- | --- | --- |
|  |  | 1 | 2 | 3 |  |
| 1 | *y* | 1 | 0 | 1 | 1 |
|  | *p* | 0.2 | 0.2 | 0.5 | 0.68 |
| 2 | *y* | 0 | 0 | 0 | 0 |
|  | *p* | 0.1 | 0 | 0.1 | 0.19 |

*References*

Hollenbeck, J. P., Saab, V. A. & Frenzel, R. W. (2011) Habitat suitability and nest survival of White-headed Woodpeckers in unburned forests of Oregon. *Journal of Wildlife Management*, **75**, 1061-1071.

Ohmann, J. L. & Gregory, M. J. (2002) Predictive mapping of forest composition and structure with direct gradient analysis and nearest-neighbor imputation in coastal Oregon, USA. *Canadian Journal of Forest Research*, **32**, 725-741.

Latif, Q. S., Saab, V. A., Mellen-Mclean, K. & Dudley, J. G. (2015) Evaluating habitat suitability models for nesting white-headed woodpeckers in unburned forest. *The Journal of Wildlife Management*, **79**, 263-273.

Appendix B. Calibration of initial population abundance.

We calibrated population abundance in monitoring year 1 by matching data from preliminary simulations to pilot data. Pilot data were collected in 2012 along the 30 transects established for regional monitoring, wherein WHWO were detected at 47 survey points along 16 transects. We varied population density (0.0664–0.5974 home ranges per 70 ha) across a series of scenarios, simulated populations for each scenario within 5 km of the 30 pilot transects, and mimicked occupancy surveys of these populations using rSPACE. As in primary simulations (see main manuscript), home ranges in these simulations were centered in suitable nesting habitat (Latif et al. 2015), space use within home ranges was not constrained by habitat, and 95% of space use occurred within 1 km of the home range center. Table B1 summarizes the number of transects and points where WHWO were detected in simulated datasets (*n* = 30 per density scenario). Simulated data most resembled pilot data for a population density of 0.2522 home ranges per 70 ha, so we set abundance in monitoring year 1 to this value when analyzing power for observing occupancy trends.

Table B1. Number of transects and points where WHWO were detected in preliminary simulations of White-headed Woodpecker regional occupancy monitoring.

| HR density (per 70 ha) | Number transect detections (95^th^ %-iles) | Number point detections (95^th^ %-iles) |
| --- | --- | --- |
| 0.0664 | 7 (3,11) | 9 (3,16) |
| 0.1726 | 15 (8,22)* | 23 (16,37) |
| 0.2257 | 17 (8,25)* | 31 (18,40) |
| 0.2389 | 19 (14,23)* | 32 (22,40) |
| 0.2522 | 20 (13,24)* | 35 (22,52)* |
| 0.2788 | 20 (17,26) | 37 (29,53)* |
| 0.385 | 24 (20,27) | 51 (41,65)* |
| 0.5974 | 28 (25,30) | 77 (63,93) |

*Indicates where simulated data matched pilot data, i.e., 95^th^ percentiles overlapped 16 and 47 transects and points, respectively.

*References*

Garrett, K. L., Raphael, M. G. and Dixon, R. D. 1996. White-headed woodpecker (*Picoides albolarvatus*). *Birds of North America*. Cornell Lab of Ornithology. Issue 252.

Latif, Q. S., Saab, V. A., Mellen-Mclean, K. & Dudley, J. G. (2015) Evaluating habitat suitability models for nesting white-headed woodpeckers in unburned forest. *The Journal of Wildlife Management*, **79**, 263-273.

Appendix C. Alternative approaches to monitoring, analysis, and inference

Supplemental details and rationale on the three monitoring approaches and other data analysis methods are provided here. For the two monitoring approaches entailing analysis of repeat-survey data with occupancy models, the potential for movement between repeat surveys meant that models estimated the probability of a given transect intersecting ≥ 1 home range, i.e., true occupancy (MacKenzie and Royle 2005, Efford and Dawson 2012). Detectability then quantified the product of availability (*p*_a_; probability of ≥ 1 individual being within range of the surveyor when surveying an occupied transect) and perceptibility (*p*_p_; the probability of ≥ 1 individual being observed assuming ≥ 1 is available for detection; Marsh and Sinclair 1989, Amundson et al. 2014, Latif et al. 2016). For simulated populations, because we assumed no observer error (*p*_p_ = 1), detectability equaled availability. With no observer error (*p*_p_ = 1), single-survey logistic regression estimates quantified the product of physical presence (ψ × *p*_a_). In reality with observer error, detection-nondetection data would also depend on observer error (P[*Y*_i_] = ψ × *p*_a_ × *p*_p_), so auxiliary sampling would be needed to inform single-survey occupancy estimates single-survey occupancy methods (e.g., double-observer or removal designs; Gorresen et al., 2008, Rota et al., 2009, Latif et al., 2016).

For occupancy models, we explicitly modeled the occupancy state of a transect (*z* ϵ {0,1}), which we assumed could change among years but not between surveys within a year (Royle and Kery, 2007). For each simulation, we compiled a two-dimensional data matrix **y**, where element *y_jt_* ϵ {0,1,2} described the number of visits at transect *j* (*j* = 1, …, *J*; *J* = number of transects) in year *t* (*t* = 1, …, 20) when white-headed woodpecker were detected. We modeled the occupancy state of a transect as

$[z_{jt}|\psi_{t}] \sim Bernoulli(\psi_{t})$,

where *ψ_t_* is the probability of transect occupancy in year *t*. We estimated occupancy probabilities separately by year on a log-odds scale:

$\mathrm{logit}\left( \psi_{t} \right)=\beta_{0,t}$.

We modeled transect detection data as binomially distributed with probability of success *p_t_* × *z_jt_*:

$\left[ y_{jt}|p_{t},z_{jt} \right]\sim Bin\left( K_{jt},p_{t}\times z_{jt} \right)$,

where *K_jt_* ϵ {1,2} is the number of surveys conducted at transect *j* in year *t*. This data structure and model formulation deviates slightly from those of others (MacKenzie et al., 2002, Tyre et al., 2003, Royle and Kery, 2007) but is functionally equivalent because *p_t_* was held constant between surveys within a given year. For the yearly-*p* model, detection probability was modeled as a fixed effect of year,

$\mathrm{logit}\left( p_{t} \right)=b_{0,t}$,

and held constant for the constant-*p* model,

$\mathrm{logit}\left( p_{t} \right)=b_{0}$.

We analyzed single-survey data using generalized linear models with binomial error, a.k.a. logistic regression (Gelman et al., 2007). We modeled the unconditional probability of detecting white-headed woodpecker on a log-odds scale with a fixed effect of year,

$\mathrm{logit}\left( {\psi'}_{t} \right)=\beta_{0,t}$),

with a data model of

$\left[ y_{jt}|{\psi'}_{t} \right]\sim Bern\left( {\psi'}_{t} \right)$.

Because we assumed no observer error, ψ’_t_ = ψ_t_ × *p*_a_ = the probability of physical presence of at least one white-headed woodpecker during a survey.

We quantified occupancy trends as the proportion yearly change in odds occupancy:

$\lambda_{\psi}=\frac{{\psi_{t+1}}/\left( 1-\psi_{t+1} \right)}{{\psi_{t}}/\left( 1-\psi_{t} \right)}$, which is functionally preferable to proportion change in occupancy (ψ*_t_*_+1_/ ψ*_t_*) because the allowable range does not depend on initial occupancy (ψ*_t_*; MacKenzie et al., 2006). Occupancy dynamics are typically modeled as a Markovian process to account for site fidelity (Royle and Kery, 2007), but doing so would have been inappropriate here given our simplifying assumption of complete site fidelity. We therefore fitted occupancy models with fixed year effects (see also Ellis et al., 2014), and then fitted a least-squares trend line to each posterior $\hat{\mathrm{logit}\left( \psi_{t} \right)}$ estimate to derive posterior estimates of $\hat{\bar{\lambda_{\psi_{t}}}}$ (hereafter $\hat{\lambda_{\psi}}$ back-transformed from $log\left( \hat{\lambda_{\psi}} \right)$). Estimated trends were not conditioned upon observed detection histories (*contra* finite-sample estimates; MacKenzie et al., 2006;Royle and Kery, 2007).

We fitted occupancy models in JAGS (Plummer 2003; operated from R via R2jags, Sturtz et al. 2005) and derived posterior trend estimates in R (for model code, see Appendix D). We fitted models using uninformative priors (truncated *Normal*[0,10]*,* min = -10, max = 10) for *logit* probabilities (*β*_0_, *b*_0_) and 4 parallel MCMC samplers, which drew posterior estimates until convergence (*n*_effective_ ≥ 100 and $\hat{R}$<1.1; Gelman and Hill, 2007). Chain lengths for samplers were initially set to 5000 and increased as needed for analyses of particular datasets to meet convergence criteria.

*Implications of assuming no observer error*

Given use of call broadcast surveys, our assumption of no observer error (*p*_p_ = 1) is plausible but unverified. By calibrating simulations with pilot data, overall detectability (perceptibility × availability) in monitoring year 1 was likely realistic. If perceptibility is in fact imperfect (*p*_p_ < 1), however, availability may be higher than represented in our simulations, i.e., home ranges may be smaller, more overlapping, or more densely distributed such that individuals are more reliably available for detection when surveying occupied transects. Explicitly calibrating simulations to more accurately reflect these parameters could yield additional insights into population ecology, but are unlikely to change our conclusions on study design and the value of single-survey approaches for monitoring regional trends.

Amundson, C. L., Royle, J. A. & Handel, C. M. (2014) A hierarchical model combining distance sampling and time removal to estimate detection probability during avian point counts. *Auk*, **131**, 476-494.

Efford, M. G. & Dawson, D. K. (2012) Occupancy in continuous habitat. *Ecosphere*, 3, article 32.

Ellis, M. M., Ivan, J. S. & Schwartz, M. K. (2014) Spatially explicit power analyses for occupancy-based monitoring of wolverine in the U.S. Rocky Mountains. *Conservation Biology,* **28,** 52-62.

Gelman, A. & Hill, J. (2007) *Data analysis using regression and multilevel/ hierarchical models*. Cambridge University Press, New York, NY.

Gelman, A., Jakulin, A., Su, Y.-S. & Pittau, M. G. (2007) A default prior distribution for logistic and other regression models. *Annals of Applied Statistics,* **2,** 1360-1383.

Gorresen, P. M., Miles, A. C., Todd, C. M., Bonaccorso, F. J. & Weller, T. J. (2008) Assessing bat detectability and occupancy with multiple automated echolocation detectors. *Journal of Mammalogy,* **89,** 11-17.

Hutto, R. L. (2016) Should scientists be required to use a model-based solution to adjust for possible distance-based detectability bias? *Ecological Applications,* **26,** 1287-1294.

Latif, Q. S., Ellis, M. M. & Amundson, C. L. (2016) A broader definition of occupancy: Comment on Hayes and Monfils. *The Journal of Wildlife Management,* **80,** 192-194.

MacKenzie, D. I., Nichols, J. D., Lachman, G. B., Droege, S., Royle, J. A. & Langtimm, C. A. (2002) Estimating site occupancy rates when detection probabilities are less than one. *Ecology,* **83,** 2248-2255.

MacKenzie, D. I., Nichols, J. D., Royle, J. A., Pollock, K. H., Baily, L. L. & Hines, J. E. (2006) *Occupancy Estimation and Modeling*. Elsevier Inc.

Marsh, H. & Sinclair, D. F. (1989) Correcting for visibility bias in strip transect aerial surveys of aquatic fauna. *The Journal of Wildlife Management,* **53,** 1017-1024.

Plummer, M. (2003) JAGS: A program for analysis of Bayesian graphical models using Gibbs sampling. *Proceedings of the 3rd International Workshop on Distributed Statistical Computing (DSC 2003)*, March 20-22, Vienna, Austria.

Rota, C. T., Fletcher Jr, R. J., Dorazio, R. M. & Betts, M. G. (2009) Occupancy estimation and the closure assumption. *Journal of Applied Ecology,* **46,** 1173-1181.

Royle, J. A. & Kery, M. (2007) A Bayesian state-space formulation of dynamic occupancy models. *Ecology,* **88,** 1813-1823.

Sturtz, S., Ligges, U. & Gelman, A. (2005) R2WinBUGS: a package for running WinBUGS from R. *Journal of Statitical Software*, 12, 1-16.

Tyre, A. J., Tenhumberg, B., Field, S. A., Niejalke, D., Parris, K. & Possingham, H. P. (2003) Improving precision and reducing bias in biological surveys: estimating false-negative error rates. *Ecological Applications,* **13,** 1790-1801.

Appendix D. Source code for initiating simulations and defining models for data analysis.

A summary of key portions of scripts used to implement simulations and analyze resulting data is available at [https://github.com/qureshlatif/WHWO-Regional-monitoring-simulation-project/ Source_code_manuscript_supplement.r](https://github.com/qureshlatif/WHWO-Regional-monitoring-simulation-project/%20Source_code_manuscript_supplement.r)

All scripts for conducting simulations and compiling and analyzing resulting data are at <https://github.com/qureshlatif/WHWO-Regional-monitoring-simulation-project>
